# Sperm Associated Antigens: Vigorous Influencers in Life

**DOI:** 10.22074/cellj.2021.7377

**Published:** 2021-10-30

**Authors:** Samaneh Faraji, Mohsen Sharafi, Abdolhossein Shahverdi, Rouhollah Fathi

**Affiliations:** 1 Department of Molecular and Cellular Biology, Faculty of Basic Science and Advanced Technologies in Biology, University of Science and Culture, ACECR, Tehran, Iran; 2 Department of Embryology, Reproductive Biomedicine Research Center, Royan Institute for Reproductive Biomedicine, ACECR, Tehran, Iran; 3 Reproductive Epidemiology Research Center, Royan Institute for Reproductive Biomedicine, ACECR, Tehran, Iran

**Keywords:** Antigen, Cancer, Fertility, Human, Sperm

## Abstract

Sperm associated antigens (SPAGs) are specific proteins in terms of performance and evolution, that have common expressions in the testes or sperm cells. Moreover, the humoral immune response against some of SPAGs can result in immunological infertilities. On the other hand, recent studies have explored several new properties of SPAGs and shed light on sperm's function, the impact of anti-sperm antibodies (ASA) in immunological infertility, and some tumors related to SPAGs. This article presents an exhaustive review of SPAGs and their roles in the cell cycle, signaling pathways, fertility, sperm-oocyte cross-talk as well as their unfavorable positions as prognostic factors in many types of cancers.

## Introduction

Sperm associated antigens (SPAGs) family comprises 18 proteins, first described as sperm membrane proteins, which could stimulate immune responses as immunogens. The molecular weight of these antigens is considered between 24-71 kDa and each protein-coding gen is located on a particular chromosome (Table 1). The role of SPAGs in the structural integrity of sperm tail, sperm motility, evaluation, fertility, cell adhesion, and cell signaling of spermatozoa has been revealed in a study by Bohring et al. (1). Silina et al. (2); also showed SPAGs 1, 2, 8, 9, 12, 13, and 15 may play roles in male infertility. About 13% of the general reproductive-age population are suffered from fertility problems, and malefactors are estimated to up to 30% of them (3). More than 90% of male infertilities are due to low count or low quality of sperm cells (4) and various factors could influence this process. Moreover, these antigens play a vigorous role in sperm and egg cross-talk and subsequent development of derived embryos. In 2017, Cui et al. (5) study showed that during sperm evolution, the level of expression for some proteins such as SPAGs 6 and 16 that impact the processes of spermatogenesis, cellular motility, energy metabolism, and oxidative phosphorylation has been increased. Since protein synthesis has been ceased throughout spermiogenesis, so most of the changes occurring at the sperm surface are a result of the sperm’s interaction with the surrounding environment (6). Any modification of these proteins may result in a change in sperm function and capabilities (7). The other factors that affect male fertility are anti-sperm antibodies. These antibodies can disrupt the power of sperm fertility and produce types of immunological infertility. Mainly one of the causes of male infertility could be due to the interaction between anti-sperm antibodies and SPAGs (8). In addition, this research has several practical applications. The most important case points out to cryopreservation and of molecular importance of healthy sperms. Using frozen healthy sperms in infertility clinics in order to artificial insemination is a case in point. The purpose of this study is to present a comprehensive review of databases and many pieces of research on SPAGs and also the result of our study on the cryopreservation of human sperm. 

### Overview of SPAGs expression status and function

SPAG proteins present in many tissues and cells such as sperm cells. According to their particular functions in sperm, any deficiency of them may lead to infertility due to sperm dysfunction. The expression of SPAGs in various organs or cells is summarised in Table 2.

The performance of these momentous proteins separately has been investigated in different studies and comprehensively compiled in this study. So far the introduction has focused on general information about SPAGs. The following section will discuss the function of each SPAG.

**Table 1:** A summary overview of the main characteristics of all sperm associated antigen (SPAG) genes


Gene name (Aliases)	Chromosome	Location	Exon count	Source (UniProt ID)

*SPAG1*	8	8q22.2	21	Q07617
*SPAG2 (UAP1)*	1	1q23.3	13	Q16222
*SPAG3 (HSPA2)*	14	14q23.3	1	P54652
*SPAG4*	20	20q11.22	13	Q9NPE6
*SPAG5*	17	17q11.2	24	Q96R06
*SPAG6*	10	10p12.2	15	O75602
*SPAG7*	17	17p13.2	8	O75391
*SPAG8*	9	9p13.3	9	Q99932
*SPAG9*	17	17q21.33	34	O60271
*SPAG10 (MFGE8)*	15	15q26.1	12	Q08431
*SPAG11B*	8	8p23.1	7	Q08648
*SPAG12 (SNU13/NHP2L1)*	22	22q13.2	6	P55769
*SPAG13 (SSFA2/KRAP)*	2	2q31.3	22	P28290
*SPAG15 (SPAM1/PH20)*	7	7q31.32	9	P38567
*SPAG16*	2	2q34	33	Q8N0X2
*SPAG17*	1	1p12	56	Q6Q759
*SPAG18 (PSMA5)*	1	1p13.3	9	P28066


The function of SPAG1 is attributed to the cytoplasmic assembly of the ciliary dynein arms, nucleotide guanosine triphosphate binding (GTP), and GTPase activity. Recent evidence suggests that the GTPase activity of SPAG1 plays a role in mammalian gametogenesis and fertility. This protein plays an important role in the oocyte via its potential association in adenosine monophosphate-activated protein kinase (AMPK) and mitogen-activated protein kinase (MAPK) signaling pathways (9). Besides, findings indicated this protein could be involved in female infertility through anti-sperm antibodies too. *SPAG1* gene indirectly inactivates phosphoinositide 3-kinase/ RAC-alpha serine/threonine-protein kinase pathway (PI3K/ AKT) which can result in inhibition of immature Sertoli cell growth via microRNA 638 (miR-638) and can lead to apoptosis (10). 

SPAG2 is part of the sperm axoneme structural element in the outer dense fiber (11). The practical roles of this protein in the cells can be attributed to the biosynthesis pathway of UDP-N-Acetyl-Alpha-D-glucosamine which is itself part of nucleotide-sugar biosynthesis. It could be noted that SPAG2 has a nucleotidyltransferase role in the cytoplasm (GeneCard ID GC01P162561).

Based on a study by Bohring et al. (1), which first described SPAG3 function as heat shock protein family A member 2 (HSPA2) according to identification by two-dimensional polyacrylamide gel electrophoresis (2D-PAGE) and matrix-assisted laser desorption/ ionization mass spectrometry (MALDI-MS); SPAG3 was assumed as HSPA2. The functional role of HSPA2 in cells can be ascribed to molecular chaperone, in a wide variety of cellular processes (UniProt ID P54652). Research by Ayaz et al. (12) has argued that induced HSPA2 by oxidative stress (ROS) has a role in male factor infertility, and any damage in sperm function can result in the reduction of *HSPA2* expression level, mitochondrial dysfunction, and induced oxidative stress in varicocele patients (13). In a study conducted by Bromfield et al. (14), it was shown that alkylation of HSPA2 with an attendant decrease in zona pellucida-receptor arylsulphatase A (ARSA) occurs during capacitation due to oxidative stress. This can destroy the zona pellucida receptor complex on sperm surface and consequently have an effect on sperm-egg cross talk. Subsequent study has unveiled that Protein Disulfide Isomerase A6 (PDIA6) is a potential HSPA2- adhering protein that is located in sperm head peri-acrosomal region and very fragile to oxidative stress (15).

SPAG4 is involved in spermatogenesis and protein localization in the sperm axoneme. The quoted protein may assist in the organization and assembly of outer dense fiber, and sperm tail-specific structure (16). It also plays a role in nuclear envelope integrity and preservation (UniProt ID Q9NPE6). Pairing performance of nucleus-cytoskeleton in spermatozoa and establishment of strength in head-tail junction complex are other functions that are clarified on SPAG4 in sperm cells (17).

**Table 2:** An overview of tissues in which sperm associated antigens (SPAGs) are expressed


SPAGs	Organs/cells	Cellular status

*SPAG1*	Lungs, Large intestine, Kidneys, Braini, Testicles, Neck, and midpiece of pachytene primary Spermatocytes (UniProt ID Q07617)	Part of the cytoskeleton microtubules in the Cytoplasm
*SPAG2/UAP1*	Testicle, Somatic tissuesii, Low expression level in Placenta, Muscle, and Liver (UniProt ID Q16222)	Cytoplasm
*SPAG3/ HSPA2*	Ubiquitous, Globus pallidusi (UniProt ID P54652; GeneCard ID GC14P064535)	Cytoskeleton and mitotic cell cycle
*SPAG4*	Testicle, Sperm cells (UniProt ID Q9NPE6)	Cytoplasmic membrane, Cytoplasm, Nucleus membrane
*SPAG5*	Testis, Placenta, Liver, Pancreas, Thymus, Colon^iii^ (UniProt ID Q96R06)	Cytoskeleton, Cytoplasm
*SPAG6*	Testis^iv^ (UniProt ID O75602)	Cytoskeleton, Flagellum
*SPAG7*	Fetal brain (UniProt ID O75391)	Nucleus
*SPAG8*	Testis germ cells^v^ (UniProt ID Q99932)	Nucleus, Cytoplasm
*SPAG9*	Testis^i^ (UniProt ID O60271)	Cytoplasm
*SPAG10/MFGE8*	Mammary epithelial cell surfacesi, Aortic media (UniProt ID Q08431)	External side of the plasma membrane, Endoplasmic reticulum lumen, can be secreted as extracellular exosome
*SPAG11B*	Caput and proximal corpus of the epididymis, Epididymal epithelium, Sperm head (UniProt ID Q08648; GeneCard ID GC08M007442)	Secretory protein^vi^
*SPAG12/ SNU13/ NHP2L1*	Ubiquitous (UniProt ID P55769)	Dense fibrillar component of the nucleolus
*SPAG13/ KRAP/SSFA2*	Pancreas, Testis (UniProt ID P28290)^i^	Plasma membrane, membrane bound arrangement with extracellular regions
*SPAG15/ SPAM1*	Testis^i^ (UniProt ID P38567)	Human sperm surface, Inner acrosomal membrane
*SPAG16*	Testis, Brain, Liver, Pancreas, Adrenal gland, Spinal cord, Heart, Thyroid, Trachea, Ovary, Kidneyvii (UniProt ID Q8N0X2)	Axoneme of the tail in sperm cells, Nucleus of post-meiotic germ cells
*SPAG17*	Testis^i^ (UniProt ID Q6Q759)	Axonemal central apparatus, Cilia-bearing cells consist of Lung, Brain, Uterus, Oviduct, Bronchial, Tracheal epithelial cells
*SPAG18/ PSMA5*	Ubiquitous (UniProt ID P28066; GeneCard ID GC01M109399)	Cytoplasm, Nucleus


^i^ ; High expression level, ^ii^; There are two isoforms of alanine--glyoxylate aminotransferase 1 (*AGX1*) and *AGX2 *for *SPAG2*, which former is numerous in testicle while the latter is more numerous than isoform one in somatic tissues, ^ⅲ^; *SPAG5* is highly expressed in testis. By contrast, it is detected at a low level in the placenta, liver, pancreas, thymus, and colon, ^ⅳ^; *SPAG6 *is highly expressed in the testis; however, it is not detected in the prostate, ovary, spleen, thymus, small intestine, colon, and peripheral blood leukocytes, ^ⅴ^; Although it is widely expressed in testis (germ cells), there is no expression in the liver, kidney, prostate, and small intestine, ^ⅵ^; *SPAG11* gene encodes several androgen-dependent and epididymis-specific secretory proteins, and ⅶ; It appears to be 5 isoforms for *SPAG16*, which isoform1 is detected in testis while isoform four has been detected in also testis and brain. Also, it is stated that isoform 4 has a low expression level in the liver, pancreas, adrenal gland, spinal cord, heart, thyroid, trachea, ovary, and kidney.

SPAG5 is an essential component of the mitotic spindle required for normal chromosome separation entry into anaphase. Moreover, it correlates with the duration of sister chromatid segregation, mitotic progression, maintenance of spindle pole architecture and may contribute to the regulation of separase activity (UniProt ID Q96R06). This protein interacts with sperm axoneme and outer dense fiber structural proteins (18), the integrity of centrosome (19), and has a role in embryonic development of testis (20).

SPAG6 is important for the structural integrity of sperm tail central apparatus and flagellar motility. In addition, it contributes to ciliogenesis in bronchial epithelium cells (21). Recent evidence suggests that SPAG6 is involved in proliferation, differentiation, a function of brain neurons (22), and the formation of the immunological synapses (23). Li et al. (22) have identified the regulation role of SPAG6 in cell apoptosis through the tumor necrosis factor-related apoptosis-inducing ligand (TRAIL) signaling pathway and fibroblast cell growth. Moreover, SPAG6 contributes to proliferation, migration, and cell morphology modulation (24). In a study by Huo et al. (25), one of the important causes of asthenospermia appears to lie in SPAG6, due to reduced expressions of solute carrier family 22 member 14 (*SLC22A14*) and *SPAG6* in spermatozoa.

SPAG7 has a molecular function of nucleic-acid binding protein (UniProt ID O75391). This protein may be a possible structural element of sperm acrosome (26). Furthermore, The quoted gene contributes to periodic fever, aphthous stomatitis, pharyngitis, and adenopathy (PFAPA) syndrome (27).

SPAG8 is one of the testis-specific proteins that is expressed during germ cell differentiation. The functional role of SPAG8 in the cell can be attributed to its role in spermatogenesis, fertility, and microtubule formation through interaction with Ran-binding protein 9 (RANBP9) (UniProt ID Q99932). According to Li et al. (28), G2/M phase regulation in the cell cycle is related to SPAG8 (possibly due to a delay in activation of Cyclin-dependent kinase 1 (CDK1) required for entry into mitosis). As highlighted by Wu et al. (29), it has been stated that this protein is involved in the acrosome reaction and sperm-zona pellucida interaction that is a vital prerequisite for successful fertilization. Besides, SPAG8 contributes to increasing actin-like protein (ACT) related transcriptional cyclic adenosine monophosphate responsive element modulator (CREM) activity during spermatogenesis. In addition, it has been declared that sperm-associated antigen 8 reacts with sera from melanoma patients (UniProt ID Q99932). 

SPAG9 is contributes to the positive regulation of the cell cycle, muscle cell, and neuron differentiation. It also involves in activation of JUN kinase activity (Jun proto-oncogene) and spermatogenesis (UniProt ID O60271). SPAG9 has been identified as a scaffold protein that ties Jun N-terminal kinase (JNK) and p38 mitogen-activated protein kinase (MAPK) transcription factors and signaling modules (30). The relationship between SPAG9 and endometrial stem cell differentiation, development of male germ cells, and also fertility has been widely investigated by Kashef, 2006 and Iwanaga, 2008 (31, 32). A recent study by Li et al. (33) has highlighted the role of SPAG9 in preventing cancer cells from reactive oxygen species (ROS) induced cell death. Furthermore, it has control over cell motility, invasion, and angiogenesis besides activating host immune responses (34).

SPAG10 has a role in apoptotic cell clearance, sperm-egg adhesion, mammary gland development, maintenance of epididymal and intestinal epithelium, promotion of vascularization, exocytosis, and also it facilitates antigen presentation (35). The quoted protein may act as cell adhesion protein to conjoin involuntary muscle to elastic fiber in arteries and contains a phosphatidylserine (PS) binding domain as well as an (ARG-GLY- ASP) motif, which enables the binding to integrin (35). SPAG10 attenuates acute renal injury caused by sepsis, inhibits neointima formation after arterial damage, and also transfers fatty acids to the placenta and it can facilitate angiogenesis and induce recovery from ischemia (36-38). The studies presented thus far provide evidence that SPAG10 promotes phagocytosis and inhibits inflammation. Besides, this protein responds to cerebral infarction by endogenous protective factors. Above and beyond, the quoted protein has a role in Alzheimer’s disease, subarachnoid hemorrhage, and prion disease (39). An overexpression of SPAG10 is correlated with several carcinomas (40).

The specific functions of SPAG11 have not been specified yet (UniProt ID Q08648). Nevertheless, it is thought that this protein is involved in the adhesion, maturation, storage, and protection of sperm. The most attractive aspect of this protein is that some of the isoforms are consist of regions that present resemblance to beta-defensin, a family of antimicrobial peptides (41). The functional role of SPAG11 in the cell can be attributed to spermatogenesis and inhibition of inflammatory parameters in rheumatoid arthritis (42).

It can be noted that SPAG12 was originally titled non-histone chromosome protein 2-like1 (NHP2L1), because of the similarities between the *SPAG12* sequence and Saccharomyces cerevisiae non-histone protein 2 (NHP2). This protein seems to be a highly conserved nuclear protein that is a component of the [U4/U6. U5] tri-snRNP that binds to the 5ˊ stem-loop of U4 snRNA. Another name of this protein is small nuclear ribonucleoprotein 13 (SNU13) involved in pre-mRNA splicing as part of the spliceosome. Moreover, it binds to the 5ˊ stem-loop of U4 snRNA and contributes to spliceosome assembly through that (UniProt ID P55769). SPAG12 has a role in fertilization and also functions in elements of the sperm tail, midpiece, and post-acrosomal region (43). Adjustment disorder in this gene has been seen in some cancers (44, 45).

SPAG13 is involved in signaling receptor and actin filament bindings. The most interesting finding of this protein is that sperm-associated antigen 13 is actively involved in energy equilibrium and obesity (46).

SPAG15 is a receptor that contributes to sperm-zona pellucida interaction. Moreover, it is a hyaluronidase that enables sperm cells to interpenetrate via hyaluronic acid-rich mass cell layer that surrounds the egg cell (UniProt ID P38567). SPAG15 is involved in sperm maturation, intracellular signaling (47), fluid reabsorption, and urine condensation in the kidney (48).

SPAG16 is appearing to lie in sperm flagella function and plays a role in motile ciliogenesis as well (UniProt ID Q8N0X2). *SPAG16* encodes 2 major proteins that associate with the axoneme of the tail in sperm cells and the nucleus of post-meiotic germ cells respectively (49). Knevel et al. (50) has demonstrated that SPAG16 protects against joint demolition in autoantibody-positive rheumatoid arthritis. The quoted protein exerts structure, stability, motility, survival, and stress-induced reaction in astrocytes and neurons of the brain. Furthermore, with the accentuate increasing expression of this protein, it is identified as the goal of autoantibodies in humoral immune responses against multiple sclerosis (MS) autoimmune disease (51).

SPAG17 is involved in axonemal central apparatus construction and epithelial cilium beating. Additionally, it may have a role in endochondral bone formation, most likely because of the primary cilia performance of chondrocytes and osteoblasts. Findings corroborate that it is localized in the central pair of the sperm flagellar axoneme and interacts with SPAG6 via the C-terminus so thereby the interaction occurs on polymerized microtubules. The quoted protein is identified in the cytoplasm of globular spermatid cells and the condensing spermatids (UniProt ID Q6Q759). Asthenozoospermia (AZS) is a prevalent cause of male infertility which is determined by abnormal reduction in motility of ejaculated spermatozoa. A recent study has pointed out a homozygous mutation in *SPAG17* through exome sequencing. Consequently, due to the fact that SPAG17 is localized in the axonemal central apparatus, it may be a new pathogenic gene-associated AZS (52). Moreover, SPAG17 has an effect on the fertility and differentiation of male germ cells (53). A report by Teves et al. (54) 2015 pointed out that deficiency of SPAG17 may result in skeletal malformations and bone deformation.

**Table 3:** A summary overview of cancers in which sperm associated antigens (SPAGs) are involved


SPAG	Cancer type

*SPAG1*	Pancreatic tumorigenesis (55), Renal cancer, Seminoma, Colon cancer, Breast cancer (2)
*SPAG2/UAP1*	Prostate cancer (56), Lymphoma, Renal cancers (2)
*SPAG3/HSPA2*	Pancreatic tumorigenesis (57)
*SPAG4*	Lung cancer, Kidney cancer (58), Prostate cancer, Liver cancer, Breast cancer (2)
*SPAG5*	Urothelial cancer (59), Breast cancer (2, 60), Lung cancer (61, 62), Gastric cancer (61), Hepatocellular carcinoma (63), Bladder cancer (2)
*SPAG6*	Myelodysplastic syndrome (24), Spinal cord neoplasm, Prostate cancer, Colon cancer (2)
*SPAG7*	Brain cancer, Synovial sarcoma, Prostate cancers (2)
*SPAG8*	Lung cancer, Breast cancer, Cervical carcinoma (2)
*SPAG9*	Thyroid cancer, Hepatocellular carcinoma, Renal cell carcinoma, Gastric cancer, Endometrial carcinoma, Lung cancer, Osteosarcoma, Breast cancer, Cervical cancers, Acute myeloid leukemia, Ovarian cancer (34), Brain cancer (2)
*SPAG10/MFGE8*	Breast cancer, Colorectal cancer (40)
*SPAG11B*	Testicular seminoma, Breast cancer (2)
*SPAG12/SNU13/ NHP2L1*	Bladder cancer, Renal carcinoma (2)
*SPAG13/KRAP/SSFA2*	Brain cancer, Colorectal cancer, Esophagus cancer (2), Lung adenocarcinoma (64), Oral squamous cell carcinoma (65)
*SPAG15/SPAM1*	Breast cancer (66), Laryngeal carcinoma, Brain cancer, Colon cancer, Lung carcinoma, Melanoma (2)
*SPAG16*	Urothelial cancer, Breast cancer (2)
*SPAG17*	Pancreatic cancer, Brain cancer (2)
*SPAG18/PSMA5*	Colorectal cancer cell lines, Chromophobe renal cell adenocarcinoma, Gastric cancer, Bladder cancer, Lung carcinoma, Head and neck cancer, Melanoma, Pulmonary neuroendocrine tumor (67)


Based on Bohring et al. (1), that defined SPAG18 functions as proteasome subunit alpha type-5 (PSMA5) according to identification by two-dimensional polyacrylamide gel electrophoresis (2D-PAGE) and matrix-assisted laser desorption/ionization mass spectrometry (MALDI-MS); SPAG18 was assumed as PSMA5. Proteasome zeta chain is another name of the protein which encodes by this gene. The quoted protein is involved in the cell cycle progression, mitosis, and proto-oncogene tyrosine-protein kinase receptor (RET) signaling pathway. Moreover, it is essential for endopeptidase activity and threonine-type endopeptidase activity. The 26S proteasome is a multi-catalytic proteinase complex that is composed of a ring-shaped 20S proteasome core and two 19S regulatory subunits. 20S core proteasome complex is associated with proteolysis of most intracellular proteins. The structure of the core is barrel-shaped, that is consists of 4 rings of 28 different subunits; two outer loops are composed of seven alpha subunits and the two inner loops are composed of seven beta subunits. The protease activity is imposed by three beta-subunits PSMB5, PSMB6, and PSMB7. PSMA5 directly interacts with the proteasome assembly chaperone PSMG1-PSMG2 heterodimer, which contributes to 20S proteasome assembly. Proteasomes are spread throughout eukaryotic cells with high concentrations, and this complex plays numerous essential roles within the cell by linking with diverse regulatory particles. It is related to two 19S regulatory particles and forms the 26S proteasome component. Nevertheless, it participates in peptides cleavage in an ATP/ Ubiquitin-dependent process through a non-lysosomal pathway deterioration of ubiquitinated proteins. The 26S proteasome is essential for the maintenance of protein equilibrium through removing misfolded or damaged proteins. SPAG18 is correlated with the proteasome activator PA200 or PA28, which is 20S proteasome mediates ubiquitin-independent protein deterioration. This type of protease activity is required in several pathways consist of spermatogenesis (20S-PA200 complex) and the major histocompatibility complex (MHC) class I peptides loading (20S-PA28 complex), which is an essential function of a modified proteasome (immunoproteasome). PSMA5 encodes part of the peptidase T1-Alpha (T1A) family, which is a 20S core alpha subunit (UniProt ID P28066; GeneCard ID GC01M109399). 

To date, Scientists have come to understand the role of SPAGs in the process of various cancers, and different studies have proven it. In this regard, scientists have been interested in SPAGs and their contribution to tumorigenesis and angiogenesis by directly studying these genes. Table 3 presents the role of all SPAGs in various cancers. According to these data, *SPAGs 9* and *5* have the highest impact on carcinogenesis, respectively. 

Hitherto, a horde of studies has revealed that various factors can have an impact on gene expression likewise sperm functions. In the past years, some evidence has been provided to explain the unfavorable influence of cryopreservation procedures on human spermatozoa. Most papers written on sperm cryopreservation include a section relating to DNA damage and epigenetic changes as a consequence of ROS (68, 69) and the detrimental impact of freezing on momentous macromolecules in particular transcriptome and proteins (70). To the best of the authors’ knowledge, no report has been found so far studying the role of the freezing procedure on these consequential proteins. Thereby, due to the importance of these proteins in the fertility process and the plentiful application of freezing in clinical methods such as fertility preservation, the effect of freezing on the expression of SPAG genes, analysis of the freezing consequences on all of SPAGs gene expression was first carried out by Faraji et al. in Royan institute (unpublished data). Refer to the study above, decreased expression of some SPAGs genes is considered as a consequence of cryopreservation. Thereby, this could be a momentous issue in the research process. However, further studies are needed to confirm this finding.

## Conclusion

Given their biological significance and roles, SPAGs appear to be a promising research vision for the future. The current approaches in literature have indicated that SPAG 2, 3, 5, 6, 9, 10, and 18 are currently the most popular sperm-associated antigens for investigating among scholars. Specifically, any changes that may occur in the sperm cells, may play a remarkable role in the development and future of the fetus. From a cautionary perspective, it could be pointed out that any alteration in these proteins may affect infant diseases and likely some diseases appear later on. Sperm-associated antigens have the ability to be changed during the freezing process. Although gene activity and expression are restricted in mature spermatozoa, they could still be altered during the freezing procedure. Cryopreservation can alter the gene expression of *SPAGs*, and it is an important issue to be concerned about, for the reason that these changes could be sustained in the future and even have repercussions on the next generation.
